# The Fluorescence Response of Four Crystalline Starches According to Ultrasound-Assisted Starch-Salicylic Acid Inclusions

**DOI:** 10.3390/foods12071431

**Published:** 2023-03-28

**Authors:** Rui Pei, Hao Lu, Fan Wang, Rongrong Ma, Yaoqi Tian

**Affiliations:** 1 State Key Laboratory of Food Science and Technology, Jiangnan University, Wuxi 214122, China; 2 School of Food Science and Technology, Jiangnan University, 1800 Lihu Road, Wuxi 214122, China

**Keywords:** starch, crystalline, salicylic acid, intermolecular hydrogen bonds, fluorescence enhancement, fluorescence

## Abstract

Fluorescence has shown its superior performance in the fields of starch physicochemical properties, starch–based materials, and the interactions of starch with small molecules. However, it has not been well explored in the fluorescence characteristics of starch. Herein, the fluorescence properties of four crystalline starches (A–type tapioca starch, B–type potato starch, C–type pea starch, and V–type starch, prepared with corn starch and stearic acid) were investigated using salicylic acid (SA) as an indicator. The results of inverted fluorescence microscopy, Fourier transform infrared spectroscopy, and thermogravimetric analysis indicated that SA could be included by starch. X–ray diffraction analysis further demonstrated that the inclusion of SA did not change the crystalline of the four crystal types of starches, which could provide a prerequisite for comparing the different fluorescence properties of the four crystal types of starches. Fluorescence enhancements of the four inclusions were 264.5 (B–type), 206 (C–type), 51.2 (V–type), and 28 (A–type). These results provide new insights for analyzing the fluorescence response of starch.

## 1. Introduction

Starch is a polymeric carbohydrate composed of glucose units connected by α-1, 4 or α-1, 6 glycosidic linkages. Starch is currently classified into A–, B–, C–, and V–types based on X–ray diffraction (XRD) patterns. A–type starch, which is mostly stored in cereal crops, has monoclinic crystals with eight water molecules among the double helices. B–type starch, which is mainly found in plant tubers and some high–amylose grain seeds, has a hexagonal shape with 36 water molecules among the double helices. C–type starch is a combination of the characteristics of A–type and B–type starches, and is naturally present in legumes and some rhizomes [1]. According to existing studies, the distribution of A–type and B–type traits in the same granule of C–type starch occurs in three ways: (a) central umbilical point with B–type inside and A–type outside; (b) central umbilical point with A–type inside and B–type outside; and (c) eccentric umbilical point with A–type and B–type in different locations [2]. V–type starch is formed by stacking a left–handed single helix mainly formed by amylose and suitable ligands in reverse parallel mode, and a few V–type starch can also be formed from amylopectin and guest [3,4]. Orthogonal, hexagonal, and tetragonal crystal systems are three forms of V–type starch crystal systems [5].

The fluorescent dye absorbs light at a certain wavelength and emits light waves at wavelengths greater than the absorbed wavelength. Fluorescence is widely used in starch–related fields due to its high sensitivity and short response time. Current studies on fluorescence in the field of starch include the preparation of starch–based fluorescent materials, the construction of fluorescent probes, and the preparation of starch–based fluorescent nanomicrospheres [6,7,8]. However, the aforementioned studies have mainly used starch as a carrier for fluorescent materials to investigate their extrinsic properties. The use of fluorescence to investigate the intrinsic response properties of the starch has yet to be explored. The existence of four crystal types of starches with different structures makes it necessary to investigate the fluorescence response of each crystal type of starch. Due to the excellent sensitivity of fluorescence, it is can transform the change of microscopic starch crystal structure into a macroscopic fluorescence effect. Wei et al. [9] demonstrated that the fluorescence response was significantly enhanced after freeze–drying potato starch included with 4–[p–(dimethylamino)styryl]–1–methylpyridinium (DASPI) dye to A–type. Therefore, there may be differences in the fluorescence properties of the different crystal types of starches. Salicylic acid (SA), a common fluorescent dye, is widely used as a fluorescent probe. Compared to DASPI, SA is smaller, which provides the possibility for SA molecules included by starch. However, the small molecules included by starch have problems such as a low embedding rate and insufficient embedding. Zhang et al. [10] and Tian et al. [11] have reported that ultrasound can improve the ability of starch to include small molecules. Furthermore, the use of ultrasonication for the preparation of inclusions is a well–established method [12,13,14]. Moreover, not only does the ultrasonication process promote mass transfer, but suitable ultrasonication conditions do not change the crystalline types of the starch [15]. Therefore, the preparation of starch–salicylic acid inclusions by ultrasonication is still desired.

In this study, tapioca starch, potato starch, and pea starch were chosen as A–type, B–type, and C–type starches, respectively. V–type starch, prepared using common corn starch and stearic acid, was selected. The inclusion of SA and different crystalline starches was carried out under proper ultrasound treatment. Inverted fluorescence microscopy, Fourier transform infrared (FTIR), and thermogravimetric (TGA) were used to characterize the inclusions formed by starch and SA (S–SA). The crystal types of the four inclusions were determined using wide–angle X–ray diffraction (XRD). The fluorescence spectrometer was used to determine the fluorescence spectra of the four inclusions. This study not only provides new insights into the differences in the fluorescence response properties of different crystal types of starch, but also shows promise for applications in identifying the structure of different crystal types of starch.

## 2. Materials and Methods

### 2.1. Materials

Cassava starch was supplied by Yuanye Biotechnology Co., Ltd. (Shanghai, China); pea starch was acquired from Fengwei Industrial Co., Ltd. (Shanghai, China); and common corn starch was obtained from Starpro Starch Co., Ltd. (Hangzhou, China). Potato starch, absolute ethanol, potassium bromide, and stearic acid were purchased from Sinopharm Chemical Reagent Co. Ltd. (Shanghai, China). DASPI (98%) was purchased from Sigma–Aldrich Life Sciences and Tech. Co., Ltd. (Shanghai, China). All chemicals and reagents used were of analytical grade unless otherwise noted.

### 2.2. Preparation of V–Type Starch

V–type starch was prepared according to the method of Sun et al. [16], with slight modifications. Specifically, 50 g of common corn starch was carefully weighed and added to 950 g of deionized water before being heated and stirred in an oil bath (DF–101S, Yuhua Instruments Co., Ltd., Gongyi, China) at 95 °C for 30 min. Stearic acid was weighed to 5 g and added slowly to preheated 50% ethanol, previously blended with the corn starch suspension. The mixture was continually stirred for 2 h, then the temperature was lowered to 50 °C and the mixture was agitated for one more hour, before being cooled to room temperature and allowed to stand for another 12 h. Absolute ethanol was added to the sediment for washing, and the mixture was centrifuged (TDL–5A, Jintan Youlian Instrument Research Institute, Changzhou, China) at 3500 r/min for 10 min at least three times until the supernatant was transparent. The precipitate was collected and dried in an oven (GZX–9146MBE, Boxun Medical Bioinstrumentation Co., Ltd., Shanghai, China) at 40 °C for 24 h, after which it was milled (Tube Mill control, IKA Equipment Co., Ltd., Guangzhou, China) and passed through a 100–mesh sieve.

### 2.3. Screening for Fluorescence Dyes

Four different crystal types of starches (A–type tapioca starch, B–type potato starch, C–type pea starch, and V–starch) at 500 mg were separately blended with 50 mL of DASPI and SA solution (0.015 mg/mL) and then sonicated for 30 min (30%, on–time: 3 s, off–time: 15 s) by an ultrasonic cell crusher (JY92–II, Scientz Biotechnology Co., Ltd., Ningbo, China). After sonication, all solutions were quantitatively diluted 100–fold as test samples. The control groups were only the two dyes and a physical mixture of four starches, and DASPI or SA with the same concentration as the experimental group. In order to enhance the fluorescence effect of the experimental group for DASPI, two experimental groups with a mass ratio (DASPI: starch) of 0.6 and 6 were set up for screening. Hitachi F–7000 was used to measure the fluorescence spectra, and the solution was thoroughly shaken prior to testing. The following parameters were set: DASPI excitation wavelength, 365 nm; SA excitation wavelength, 297 nm; excitation and emission slits, 5 nm; photomultiplier voltage, 700 V; scanning speed, 1200 nm/min; and emission wavelength range was 385–700 nm for DASPI and 300–550 nm for SA. Measurements were conducted in a light–proof environment.

### 2.4. The Formation of Inclusions of Starch with SA

Each of the four crystal types of starch was weighed to 500 mg and placed in four 50 mL beakers, respectively. SA (50 mL), at a concentration of 1 × 10^−4^ mol/L, was then poured into these four beakers. The mixtures were placed in an ultrasonic cell crusher for sonication with the following sonication parameters: 30% power, on–time 3 s, off–time 15 s, and 30 min sonication time. The resulting mixture was transferred to a 500 mL brown volumetric flask, and the beakers were washed several times with deionized water. The washing solution was then transferred to a volumetric flask for volume fixation, and 10 mL was carefully transferred to a 100 mL brown volumetric flask for volume fixation for quantitative fluorescence intensity measurement. The control groups were a physical mixture of A–, B–, C–, and V–type starch and SA, and the same concentration of SA. The treatment of the control group was the same as that of the experimental group, except for the ultrasound. After each test, the cuvette was washed thrice with anhydrous ethanol and water. Furthermore, sonicated A–type starch and SA, B–type starch and SA, and C–type starch and SA were extracted via a 0.45 μm microporous filter membrane and washed several times with deionized water until the filtrate was free of distinctive emissions. The sonicated V–type starch and SA were washed three times (9000 rpm, 10 min) with deionized water by centrifugation (5804R, Eppendorf AG, Hamburg, Germany). Four washed inclusions were placed in a 40 °C oven for 24 h. They were then milled into a powder (Tube Mill control, IKA Equipment Co., Ltd., Guangzhou, China) and used as samples for inclusion behavior analysis.

### 2.5. Inclusion Behavior Analysis

#### 2.5.1. Inverted Fluorescence Microscope

A small amount of each of the four prepared inclusions and four crystal types of starches was evenly spread on a slide to form a thin layer before being placed on a carrier table for observation using an inverted fluorescence microscope (Axio Vert A1, Carl Zelss Co., Ltd., Shanghai, China) with a 20× objective and a DAPI excitation filter. Fluorescence was detected in the dark field after an appropriate field of vision was found in the bright field, and the entire operation was performed under light–proof conditions.

#### 2.5.2. FTIR

The physical mixture of starch and SA (S–SA–P), four inclusions, four crystal types of starches, and SA were added to dried potassium bromide powder at a ratio of 1:100 in an agate mortar and ground well. The pressed tablets of potassium bromide were produced using a tablet press machine as test samples. An infrared lamp was used throughout the process. The sample spectra were recorded using Fourier transform infrared spectroscopy (Nicolet IS10, Thermo Fisher Scientific, Waltham, MA, USA) in the wavenumber range of 4000 cm^−1^ to 400 cm^−1^ with a resolution of 4 cm^−1^ and a total of 32 scans.

#### 2.5.3. DSC

Each starch sample (3 mg) was weighed and placed in an aluminum pan. Deionized water (6 μL) was added, and the tablets were pressed and placed in the refrigerator to equilibrate overnight. The above samples were tested by DSC (X–DSC 7000, Hitachi, Co., Ltd., Tokyo, Japan) at a heating rate of 10 °C/min from 20 to 100 °C, with an empty pan as a control. The system was calibrated with indium. The parameters recorded are gelatinization temperatures (onset (T_o_), peak (T_p_), and conclusion (T_c_) temperature) and enthalpy change (ΔH). The experiment was repeated at least three times.

#### 2.5.4. TGA

A thermogravimeter (TGA2, Mettler Toledo Co., Ltd., Zurich, Switzerland) was employed to investigate the thermodynamic degradation properties of the four inclusions, corresponding physical mixtures, four crystal types of starches, and SA at a heating rate of 10 °C/min under nitrogen atmosphere (flow rate 50 mL/min). A ceramic pan was loaded with approximately 3 mg of sample, and the temperature was increased from 50 to 600 °C.

#### 2.5.5. XRD

An X–ray diffractometer (D2 PHASER, Bruker–AXS, Karlsruhe, Germany) was used to analyze the diffraction patterns of the four inclusions, corresponding physical mixtures, four crystal types of starches, and SA. The operating voltage and current of the instrument are 30 kV and 10 mA, respectively. Diffractograms were recorded at room temperature in the 2θ angle region from 5° to 35° with a step size of 0.03° and a scan speed of 3°/min.

### 2.6. Fluorescence Spectroscopy

The fluorescence spectra of the four inclusions, corresponding physical mixture, four crystal types of starches, and SA were obtained separately using a fluorescence spectrophotometer (F–7000, Hitachi, Co., Ltd., Tokyo, Japan). The fluorescence intensity at 402 nm emission wavelength was recorded and the solutions were shaken well before each measurement. The parameters were set as follows: excitation wavelength, 297 nm; emission wavelength range, 300–550 nm; scanning speed, 1200 nm/min; excitation and emission slit, 5 nm; and photocell multiplication voltage (PMT), 700 V. Experimental and control samples were prepared and tested under light–proof conditions.

### 2.7. Reflection of Fluorescence Enhancement of Four Crystal Types of Starches

The extent of the fluorescence enhancement of the four inclusions was determined using Equation (1):

(1) $$\Delta F=F_{1}-F_{2}$$

where *F*_1_ and *F*_2_ represent the fluorescence intensities of the four inclusions and SA, respectively. The difference between the former and latter, that is, Δ*F*, represents the extent of fluorescence enhancement.

### 2.8. Statistical Analysis

Data are presented as mean ± standard deviation, and each group of trials was repeated three times before being evaluated using one–way ANOVA. ORIGIN PRO 2021 was used to perform one–way ANOVA on the data.

## 3. Results and Discussion

### 3.1. Screening of Fluorescent Dyes

**Figure 1:**
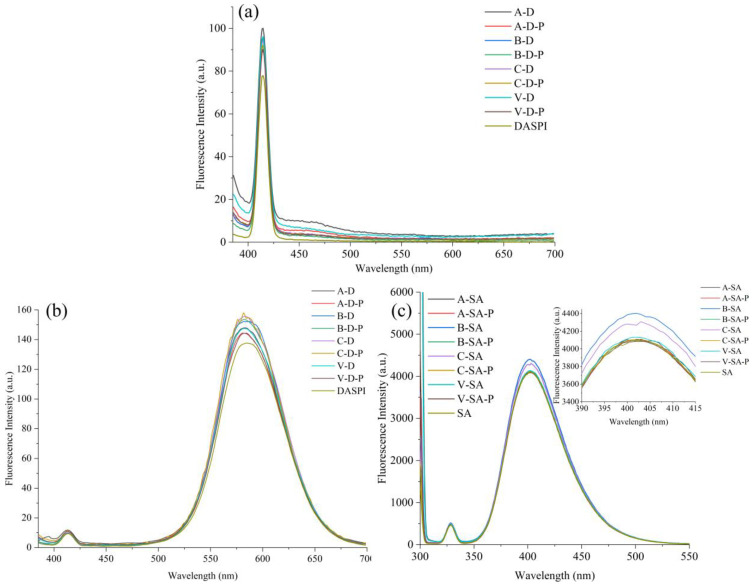
Screening of the fluorescence spectra of (**a**,**b**) DASPI and (**c**) SA dyes. (**a**,**b**) The solutions after sonication of A–, B–, C–, and V–type starches and DASPI are labeled A–D, B–D, C–D, and V–D, respectively. The physical mixtures of A–, B–, C–, V–type starch, and DASPI are labeled A–D–P, B–D–P, C–D–P, and V–D–P, respectively. (**c**) A–SA, B–SA, C–SA, and V–SA represent the inclusions of A–, B–, C–, and V–type starch, and SA after ultrasonic treatment, respectively. A–SA–P, B–SA–P, C–SA–P, and V–SA–P represent physical mixtures of the four crystal types of starches and SA, respectively.

The fluorescence spectra of the experimental and control groups in the presence of DASPI and SA are shown in Figure 1a–c, respectively. Figure 1a,b reveal that fluorescence peaks arose at 413 nm in the experimental group, the physical mixture of DASPI and the four crystal types of starches, and DASPI. However, this is a Raman scattering peak induced by the bending and vibration of the solvent water molecules [17] and not a DASPI dye peak. The DASPI dye emits fluorescence at 585 nm under 365 nm excitation wavelengths. All the curves in Figure 1a at 585 nm do not show a fluorescence peak. The fluorescence response at 585 nm is barely enhanced in all experimental groups compared to the control group in Figure 1b. However, the fluorescence response of the four inclusions at 402 nm in Figure 1c is significantly higher than that of SA and the physical mixture of SA and the four crystal types of starches, respectively. Therefore, SA is selected as the fluorescent guest.

### 3.2. Inclusion Behavior Analysis

#### 3.2.1. Inverted Fluorescence Microscope

**Figure 2:**
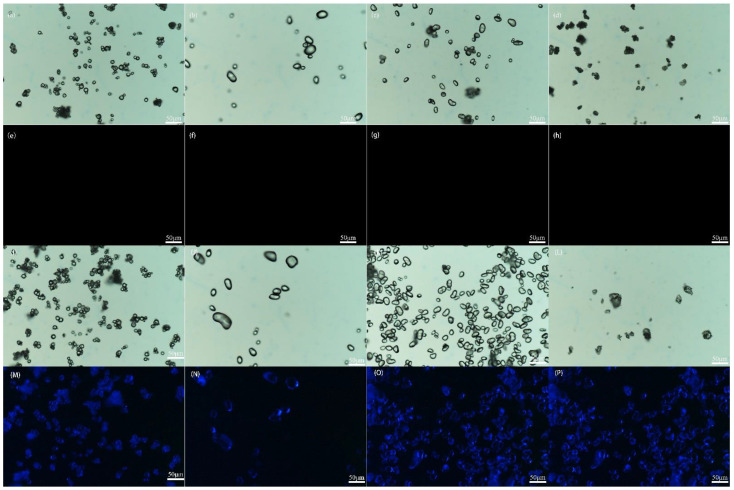
Fluorescence micrographs of four inclusions and four crystal types of starches. (**a**–**h**) represent the four unmodified starches, where (**a**–**d**) are taken in the bright field and (**e**–**h**) are taken in the dark field. A–, B–, C–, and V–type are illustrated from left to right. (**I**–**P**) represent the four inclusions, where I–L are taken in the bright field and M–P are taken in the dark field. A–SA, B–SA, C–SA, and V–SA are illustrated from left to right.

Figure 2 shows photographs taken under a fluorescence microscope with four inclusions and four unmodified starches. The four inclusions have brighter fluorescence in the dark field than in the bright field. Considering that current research shows that natural starch granules do not show fluorescence, [6,18] as e–h indicate, and that the fluorescence response of the four inclusions is stronger than that of the corresponding control group in Figure 1c, it is safe to assume that SA is included by starch. Furthermore, almost all the granules of the four inclusions fluoresce brightly. This shows that ultrasonic treatment is able to result in sufficient contact between starch and SA.

#### 3.2.2. FTIR

**Figure 3:**
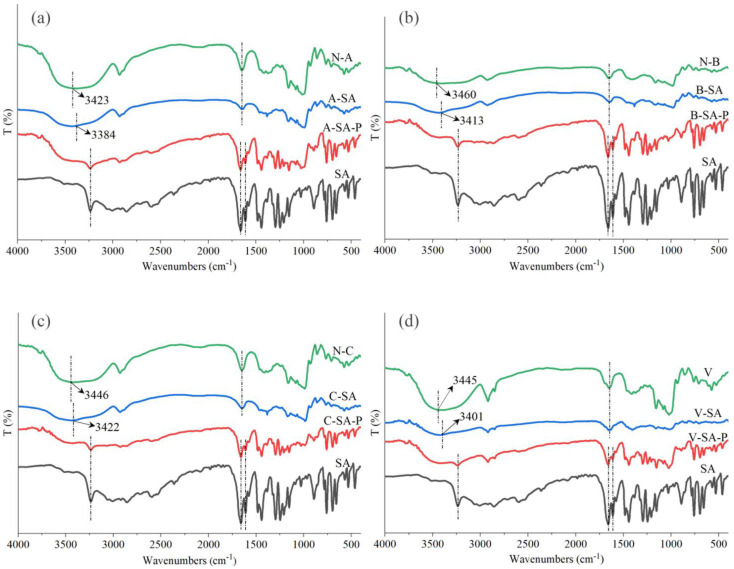
FTIR spectra of (**a**) N–A, A–SA, A–SA–P, and SA; (**b**) N–B, B–SA, B–SA–P, and SA; (**c**) N–C, C–SA, C–SA–P, and SA; and (**d**) V, V–SA, V–SA–P, and SA.N––A, N––B, N––C, and V represent four crystal types of starches, respectively. A––SA, B––SA, C––SA, and V––SA represent the inclusions of A––, B––, C––, and V––type starch, and SA after ultrasonic treatment, respectively. A––SA––P, B––SA––P, C––SA––P, and V––SA––P represent physical mixtures of the four crystal types of starches and SA, respectively.

The FTIR spectra of the four inclusions, corresponding physical mixtures, four crystal types of starches, and SA are shown in Figure 3. SA has six distinct peaks: intramolecular hydrogen bonding vibration at 3239 cm^−1^, intermolecular hydrogen bonding vibration at 2856 cm^−1^, carboxyl group vibration at 2597 cm^−1^, carbonyl group vibration at 1664 cm^−1^, C=C in the benzene ring vibration at 1614 cm^−1^, and phenolic hydroxyl group vibration at 1480 cm^−1^ [19]. The FTIR spectra of S–SA–P in Figure 3 reveal both starch and SA characteristic peaks at 3239, 1664, and 1614 cm^−1^, showing that the physical mixtures were a simple superposition of starch and SA. However, in the FTIR spectra of the four inclusions, the distinctive bands of SA at 3239, 2597, and 1614 cm^−1^ vanished, indicating that SA was successfully included by starch. In addition, the hydroxyl bands of the four inclusions were shifted from 3423, 3460, 3446, and 3445 cm^−1^ to 3384, 3413, 3422, and 3401 cm^−1^, respectively, compared to that of the four crystal types of starches. This indicates that the included SA may form hydrogen bonds with the hydroxyl groups of the four crystal types of starches.

#### 3.2.3. DSC

Table 1 presents the DSC parameters of the four crystal types of starches, the four crystal types of starches treated with ultrasound, and the four inclusions. The paste temperature parameters of the four crystal types of starches treated by ultrasonication were lower than those of the corresponding unmodified starches, which might be attributed to the thermal stability reduction of the natural starches after ultrasonic treatment. The enthalpy values of U–A, U–B, U–C, and U–V were all lower than those of the unmodified starch. This phenomenon might be related to the disruption of the partial hydrogen bonds between the starch chains by ultrasonic treatment, which is consistent with previous research [20]. However, the enthalpy values of all four inclusions were higher than those of the four crystal types of starches treated with ultrasound. The formation of hydrogen bonds between SA and starch molecules causes a decrease in the energy of the inclusion system, leading to an increase in the energy requirement for the gelatinization of the inclusions.

#### 3.2.4. TGA

**Figure 4:**
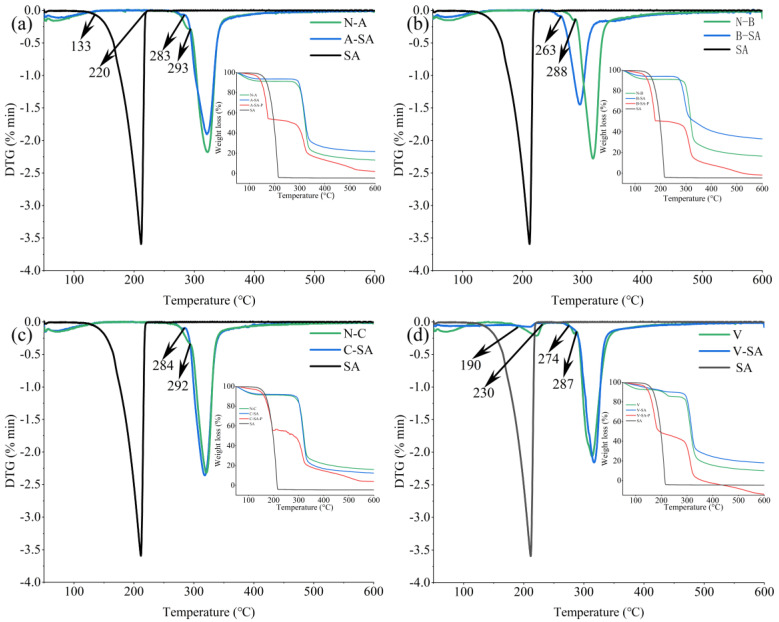
DTG curves of the four crystal types of starches, four inclusions, and SA. The insets are the TGA curves of (**a**) the thermogravimetric analysis curves of A–SA, A–SA–P, N–A, and SA; (**b**) the thermogravimetric analysis curves of B–SA, B–SA–P, N–B, and SA; (**c**) the thermogravimetric analysis curves of C–SA, C–SA–P, N–C, and SA; (**d**) the thermogravimetric analysis curves of V–SA, V–SA–P, V, and SA.

Figure 4 depicts the DTG curves of the four inclusions, four crystal types of starches, and SA. The insets show the corresponding thermogravimetric (TGA) curves. The thermogravimetric degradation curves of the physical mixture showed three typical steps, which combined the unmodified starch with the SA thermal degradation process, indicating that starch and SA existed independently in S–SA–P. However, the major heat degradation stages of the four inclusions were significantly different from those of SA and the corresponding physical mixture. This may have been caused by the formation of inclusions. Furthermore, when the inclusions were formed, the degradation temperatures of the four inclusions in the second stage decreased from 293, 288, 292, and 287 to 283, 263, 284, and 274 °C, respectively, as shown in Figure 4. Because the degradation temperature of SA ranges from 133 to 220 °C, this may pull down the degradation temperature of the inclusions in the second stage. In addition, it was noted that the V–SA degradation curve contained an additional stage induced by embedded stearic acid in the inner cavity of helices, resulting in a partial stearic acid degradation process during thermal degradation at 190–230 °C, which is consistent with the findings of previous studies [21].

#### 3.2.5. XRD

**Figure 5:**
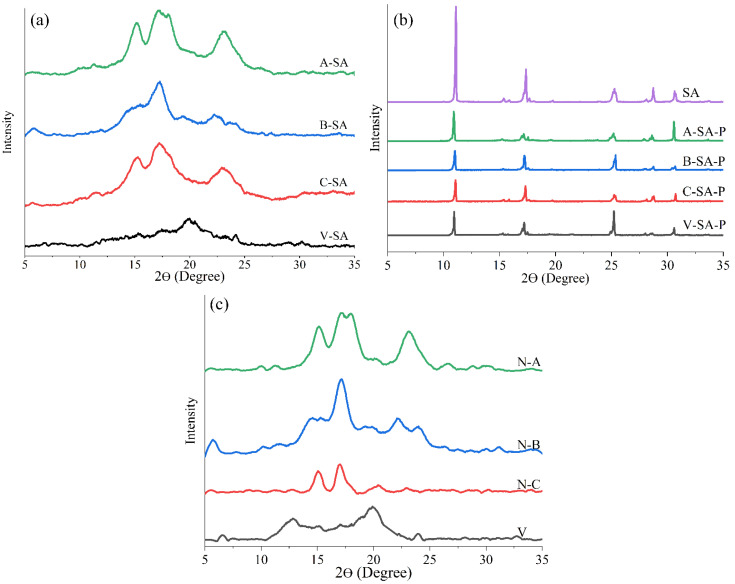
X–ray patterns of the (**a**) four inclusions, (**b**) SA and corresponding physical mixtures, and (**c**) four crystal types of native starches.

The crystal types of starches were identified by XRD. Figure 5a,b showed XRD diffraction patterns of four inclusions, and the physical mixtures of four crystal types of starches and SA, respectively. Figure 5a shows four characteristic peaks for A–SA (15°, 17°, 18°, and 23°), four characteristic peaks for B–SA (5.6°, 17°, 22°, and 24°), four characteristic peaks for C–SA (5.6°, 15°, 17°, and 23°), and three characteristic peaks for V–SA (7.5°, 12.2°, and 20°), but no characteristic peaks for SA. This may be attributed to the successful inclusion of salicylic acid by starch. In contrast, the XRD patterns of the physical mixture in Figure 5b show clear peaks of SA. The differences between these two graphs indicate that the inclusions are not a simple physical mixture of starch and SA. Furthermore, the characteristic peaks of these four inclusions were compatible with that of the four crystal types of starches, indicating that the inclusion of SA did not modify the crystal types of the four starches. The above analysis not only shows that the sonication treatment had no effect on the crystalline of the four starches, which is consistent with previous research [22,23,24], but also lays the groundwork for comparing the different fluorescence responses of the four crystal types of starches.

### 3.3. Fluorescence Spectra Analysis

**Figure 6:**
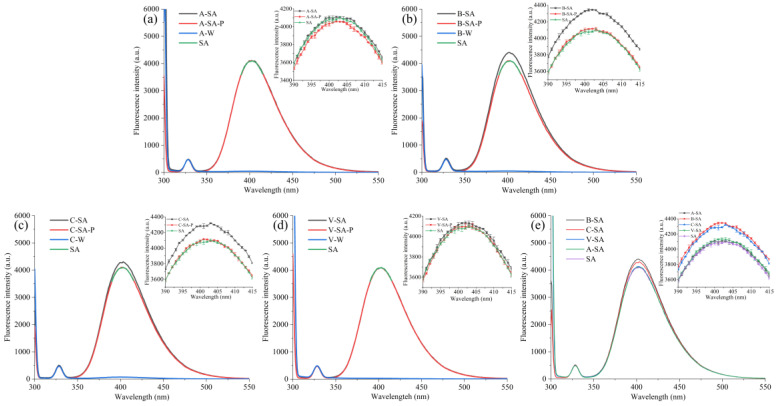
Fluorescence spectra of the four inclusions, corresponding physical mixture, four crystal types of starches, and SA. (**a**) the fluorescence spectra of A–SA, A–SA–P, A–W, and SA. (**b**) the fluorescence spectra of B–SA, B–SA–P, B–W, and SA. (**c**) the fluorescence spectra of C–SA, C–SA–P, C–W, and SA. (**d**) the fluorescence spectra of V–SA, V–SA–P, V–W, and SA. (**e**) the fluorescence spectra of A–SA, B–SA, C–SA, V–SA, and SA. All insets are local magnification of the fluorescence spectrum from 390 to 415 nm. A–W, B–W, C–W, and V–W represent the mixtures of the four crystal types of starches and water, respectively.

Figure 6 depicts the fluorescence spectra of the four inclusions, physical mixture, SA, and four crystal types of starches at 300–550 nm, with an inset showing the fluorescence spectra at 390–416 nm. According to the insets, the fluorescence intensity at 402 nm of the four inclusions was higher than that of the corresponding physical mixture, four crystal types of starches, and SA, indicating that the formation of inclusions can enhance the fluorescence intensity. Li et al. [25] reported that intermolecular hydrogen bonds can enhance the rigidity of molecular structures. This may be due to the formation of the hydrogen bonding between starch and SA, which strengthens the structural steel of the SA molecule and thus enhances the fluorescence response of the inclusions.

In addition, it should be noted that the Δ*F* values of the four inclusions listed in Table 2 are significantly different, except for A–SA and V–SA. This may be related to the difference in the helical arrangement of the structure of the four starch crystal types. A–SA has the smallest Δ*F* of the four inclusions. This might be due to the fact that the tightest arrangement of the A–type starch helix is unfavorable for the inclusion of SA, making the fluorescence response enhancement ineffective. In contrast to A–SA, B–SA exhibits the largest Δ*F*. The helix arrangement of the B–type starch is the loosest and most favorable for SA inclusion. Therefore, the B–type starch might include more SA than the other three crystal types of starches. The Δ*F* value of C–SA is second only to that of B–SA. The pea starch used in this experiment is C–type starch. The C–type starch contains both A–type and B–type crystals. This might explain why Δ*F* of C–SA is lower than that of B–SA. The Δ*F* of V–SA is smaller than that of C–SA. This could be due to the tighter arrangement of the V–type starch helix than that of the B–type. Although Δ*F* of V–SA is greater than that of A–SA, there is no significant difference between them. This might be because both A–type and V–type starches have a more compact helical arrangement. However, it is currently uncertain how much more compact they are. Putaux et al. [26] reported that the polar head of fatty acids in V–type starch is on the outer side of the helices. This may increase the resistance of the V–type starch including SA during the sonication process, making the fluorescence response of V–SA not significantly different from that of A–SA. Considering that starches of the same crystal type have a similar helical arrangement, the fluorescence response can be used to rapidly identify the starch crystal type.

## 4. Conclusions

In this study, SA was selected as the most suitable fluorescent dye. Inverted fluorescence micrographs, FTIR, and TGA demonstrated that SA was successfully included by the four crystal types of starches after sonication. The XRD analysis further indicated that the included SA did not change the starch crystal type. The inclusion of SA by the four crystalline starches enhanced the fluorescence response. At 402 nm, Δ*F* was greatest for B–, C–, V–, and A–type, in descending order, and there was a significant difference in the fluorescence response between the A–, B–, and C–type. These findings provide new insights for analyzing the fluorescence response of different crystal types of starch.

## Figures and Tables

**Table 1:** DSC parameters for natural A–, B–, C–, V–type starches (N–A, N–B, N–C, V), ultrasonicated A–, B–, C–, V–type starches (U–A, U–B, U–C, U–V), and inclusions of four crystal types of starches with salicylic acid (A–SA, B–SA, C–SA, V–SA).

	T_o_	T_p_	T_c_	ΔH
N–A	64.078 ± 0.55 ^cd^*	71.567 ± 0.96 ^a^	80.753 ± 0.18 ^a^	10.54 ± 0.24 ^ab^
U–A	62.66 ± 0.10 ^d^	69.102 ± 0.77 ^bc^	78.81 ± 1.30 ^ab^	9.42 ± 0.10 ^b^
A–SA	63.056 ± 0.08 ^d^	69.491 ± 0.76 ^b^	79.485 ± 0.18 ^ab^	10.06 ± 0.10 ^ab^
N–B	63.097 ± 0.057 ^d^	68.972 ± 0.36 ^bc^	74.990 ± 0.53 ^bc^	11.08 ± 0.12 ^a^
U–B	60.579 ± 0.23 ^e^	64.160 ± 0.01 ^d^	71.919 ± 0.97 ^c^	10.46 ± 0.44 ^ab^
B–SA	61.215 ± 0.05 ^e^	64.824 ± 0.07 ^d^	72.769 ± 1.56 ^c^	10.63 ± 0.49 ^ab^
N–C	64.773 ± 0.05 ^c^	71.465 ± 0.27 ^a^	77.162 ± 0.63 ^b^	6.99 ± 0.40 ^c^
U–C	63.341 ± 0.24 ^d^	70.017 ± 0.13 ^ab^	73.942 ± 0.55 ^bc^	5.44 ± 0.0007 ^d^
C–SA	63.975 ± 0.99 ^cd^	70.225 ± 0.24 ^ab^	76.594 ± 0.28 ^b^	6.24 ± 0.39 ^cd^
V	69.883 ± 0.03 ^a^	70.979 ± 0.01 ^ab^	74.225 ± 0.61 ^bc^	9.103 ± 0.43 ^b^
U–V	66.81 ± 0.06 ^b^	67.504 ± 0.30 ^c^	68.058 ± 0.02 ^c^	0.042 ± 0.0007 ^f^
V–SA	69.085 ± 0.29 ^ab^	70.34 ± 0.54 ^ab^	72.465 ± 1.62 ^c^	3.601 ± 0.41 ^e^

* Values are represented as the mean ± SD. Values with the same superscript letter indicate no significant differences, whereas those with different letters represent the opposite (*p* < 0.05).

**Table 2:** Enhancement of fluorescence intensity of the four inclusions (A–SA, B–SA, C–SA, and V–SA) in comparison to SA.

Inclusions	*F*_1_	*F*_2_	Enhancement Fluorescence Intensity Δ*F*
B–SA	4352.5	4088	264.5 ± 1.4 ^a^*
C–SA	4294	4088	206.0 ± 7.8 ^b^
V–SA	4139.2	4088	51.2 ± 16.3 ^c^
A–SA	4116	4088	28.0 ± 7.8 ^c^

* Values are represented as the mean ± SD. Values with the same superscript letter indicate no significant differences, whereas those with different letters represent the opposite (*p* < 0.05).

## Data Availability

The data are contained within the article.

## References

[1] Perez S., Bertoft E. (2010). The molecular structures of starch components and their contribution to the architecture of starch granules: A comprehensive review. Starch–Stärke.

[2] He W., Wei C.X. (2017). Progress in C–type starches from different plant sources. Food Hydrocoll..

[3] Li Q., Shi S.H., Dong Y.Y., Yu X.Z. (2020). Characterisation of amylose and amylopectin with various moisture contents after frying process: Effect of starch–lipid complex formation. Int. J. Food Sci. Technol..

[4] Guo J.Y., Ziegler G.R., Kong L.Y. (2022). Polymorphic transitions of V–type amylose upon hydration and dehydration. Food Hydrocoll..

[5] Le C.A.K., Choisnard L., Wouessidjewe D., Putaux J.L. (2018). Polymorphism of crystalline complexes of V–amylose with fatty acids. Int. J. Biol. Macromol..

[6] Dong X.Y., Niu X.Q., Zhang Z.Y., Wei J.S., Xiong H.M. (2020). Red fluorescent carbon dot powder for accurate latent fingerprint identification using an artificial intelligence program. ACS Appl. Mater. Interfaces.

[7] Singh A., Guleria A., Neogy S., Rath M.C. (2020). UV induced synthesis of starch capped CdSe quantum dots: Functionalization with thiourea and application in sensing heavy metals ions in aqueous solution. Arab. J. Chem..

[8] Li H.C., Zhang B., Lü S.Y., Ma H.Y., Liu M.Z. (2018). Synthesis and characterization of a nano fluorescent starch. Int. J. Biol. Macromol..

[9] Wei Y.H., Lin X.Q., Wei C., Zhang W., Yan Y.L., Zhao Y.S. (2017). Starch–based biological microlasers. ACS Nano.

[10] Zhang Z.W., Zhao F., Meng Y.L., Lin J.Z., Xu Y.P., Feng Y., Ding F., Li P.W. (2022). Microencapsulation of the Enzyme Breaker by Double–Layer Embedding Method. SPE J..

[11] Tian S.Q., Xue X.A., Wang X.W., Chen Z.C. (2022). Preparation of starch–based functional food nano–microcapsule delivery system and its controlled release characteristics. Front. Nutr..

[12] Li J., Tian L., Fang Y., Chen W., Hunag G. (2021). Ultrasonic-Assisted Preparation of Maize Starch–Caffeic Acid Complex: Physicochemical and Digestion Properties. Starch-Stärke.

[13] Mallakpour S., Nezamzadeh Ezhieh A. (2018). Preparation and characterization of starch nanocomposite embedded with functionalized MWCNT: Investigation of optical, morphological, thermal, and copper ions adsorption properties. Adv. Polym. Technol..

[14] Zhang S., Zhou Y.B., Jin S.S., Meng X., Yang L.P., Wang H.S. (2017). Preparation and structural characterization of corn starch–aroma compound inclusion complexes. J. Sci. Food Agric..

[15] Chemat F., Khan M.K. (2011). Applications of ultrasound in food technology: Processing, preservation and extraction. Ultrason. Sonochem..

[16] Sun S.L., Jin Y.Z., Hong Y., Gu Z.B., Cheng L., Li Z.F., Li C.M. (2021). Effects of fatty acids with various chain lengths and degrees of unsaturation on the structure, physicochemical properties and digestibility of maize starch–fatty acid complexes. Food Hydrocoll..

[17] Hu Y.T., Liu C., Wang X.P., Zhao D.D. (2018). Adaptive handling of Rayleigh and Raman scatter of fluorescence data based on evaluation of the degree of spectral overlap. Spectrochim. Acta Part A Mol. Biomol. Spectrosc..

[18] Wang C., He X.W., Huang Q., Fu X., Luo F.X., Li L. (2013). Distribution of octenylsuccinic substituents in modified A and B polymorph starch granules. J. Agric. Food Chem..

[19] Sun W.J., Yang X.W., Zhang H.G., Zhu L., Gao S.L. (2006). Spectra of rare earth complexes with salicylate. Acta Photonica Sin..

[20] Hu A.J., Li Y., Zheng J. (2019). Dual–frequency ultrasonic effect on the structure and properties of starch with different size. LWT–Food Sci. Technol..

[21] Chen Z., Cao L., Shan F., Fang G.Y. (2013). Preparation and characteristics of microencapsulated stearic acid as composite thermal energy storage material in buildings. Energy Build..

[22] Rahaman A., Kumari A., Zeng X.A., Adil Farooq M., Siddique R., Khalifa I., Siddeeg A., Ali M., Faisal Manzoor M. (2021). Ultrasound based modification and structural–functional analysis of corn and cassava starch. Ultrason. Sonochem..

[23] Zhu J., Li L., Chen L., Li X.X. (2012). Study on supramolecular structural changes of ultrasonic treated potato starch granules. Food Hydrocoll..

[24] Raza H., Ameer K., Ren X.F., Liang Q.F., Chen X.X., Chen H.X., Ma H.L. (2021). Physicochemical properties and digestion mechanism of starch–linoleic acid complex induced by multi–frequency power ultrasound. Food Chem..

[25] Li K., Yang L., He H.C., Liu K. (2022). Aramid–film pH sensitive fluorescence enhancement based on benzimidazole intermolecular hydrogen bonds. Opt. Mater..

[26] Putaux J.L., Nishiyama Y., Mazeau K., Morin M., Cardoso M.B., Chanzy H. (2011). Helical conformation in crystalline inclusion complexes of V–amylose: A historical perspective. Macromol. Symp..

